# Multipotent mesenchymal stromal cells play critical roles in hepatocellular carcinoma initiation, progression and therapy

**DOI:** 10.1186/s12943-018-0926-6

**Published:** 2018-12-28

**Authors:** Zeli Yin, Keqiu Jiang, Rui Li, Chengyong Dong, Liming Wang

**Affiliations:** 1 Division of Hepatobiliary and Pancreatic Surgery, Department of General Surgery, The Second Affiliated Hospital of Dalian Medical University, 467 Zhongshan Road, Dalian, 116027 Liaoning China; 2 Engineering Research Center for New Materials and Precision Treatment Technology of Malignant Tumors Therapy, Dalian Medical University, Dalian, 116027 Liaoning China; 3 Engineering Technology Research Center for Translational Medicine, Dalian Medical University, Dalian, 116027 Liaoning China

**Keywords:** Mesenchymal stromal cells (MSCs), Hepatocellular carcinoma (HCC), Chemotaxis, Carcinogenesis, Neoplasm metastasis, Tumor-targeted therapy

## Abstract

Hepatocellular carcinoma (HCC) is the most common type of primary liver cancer, with high morbidity, relapse and mortality rates. Multipotent mesenchymal stromal cells (MSCs) can be recruited to and become integral components of the HCC microenvironment and can influence tumor progression. This review discusses MSC migration to liver fibrosis and the HCC microenvironment, MSC involvement in HCC initiation and progression and the widespread application of MSCs in HCC-targeted therapy, thus clarifying the critical roles of MSCs in HCC.

## Background

Primary liver cancer is lethal and is substantially more common in men than in women. In men, it was the third-leading cause of cancer death worldwide in 2002, and the five-year survival rate was almost zero [1]. A decade later, liver cancer mortality in men has surpassed that of stomach cancer, becoming the second-leading cause of cancer deaths after lung cancer [2]. Although primary liver cancer includes hepatocellular carcinoma (HCC), intrahepatic cholangiocarcinoma (ICC) and combined hepatocellular-cholangiocarcinoma (cHCC-CC) according to histology, HCC comprises more than 90% of primary liver cancers and is thus a major histological type [3]. Despite recent advances in the prevention, surveillance, diagnosis, treatment and multidisciplinary collaboration of HCC, it remains highly lethal. Death among HCC patients occurs mainly due to tumor progression, with recurrence and metastasis, even after curative treatments at the early stage, such as resection, liver transplantation and radiofrequency ablation [4, 5]. Patients with advanced HCC have few treatment options, which include the first-line agent sorafenib [6, 7] and the second-line agent regorafenib [8]. In a recent randomized phase 3 noninferiority trial, lenvatinib was noninferior to sorafenib in overall survival in untreated advanced HCC and may be a new treatment option for advanced HCC [9]. Although these molecular targeted therapeutic drugs may prolong the survival of advanced HCC patients to some degree, the liver function requirements of these therapies limit their use in patients with severe hepatic dysfunction. Therefore, determining the mechanism of recurrence and metastasis and exploring new systemic treatment methods for HCC is of great importance.

**Fig. 1:**
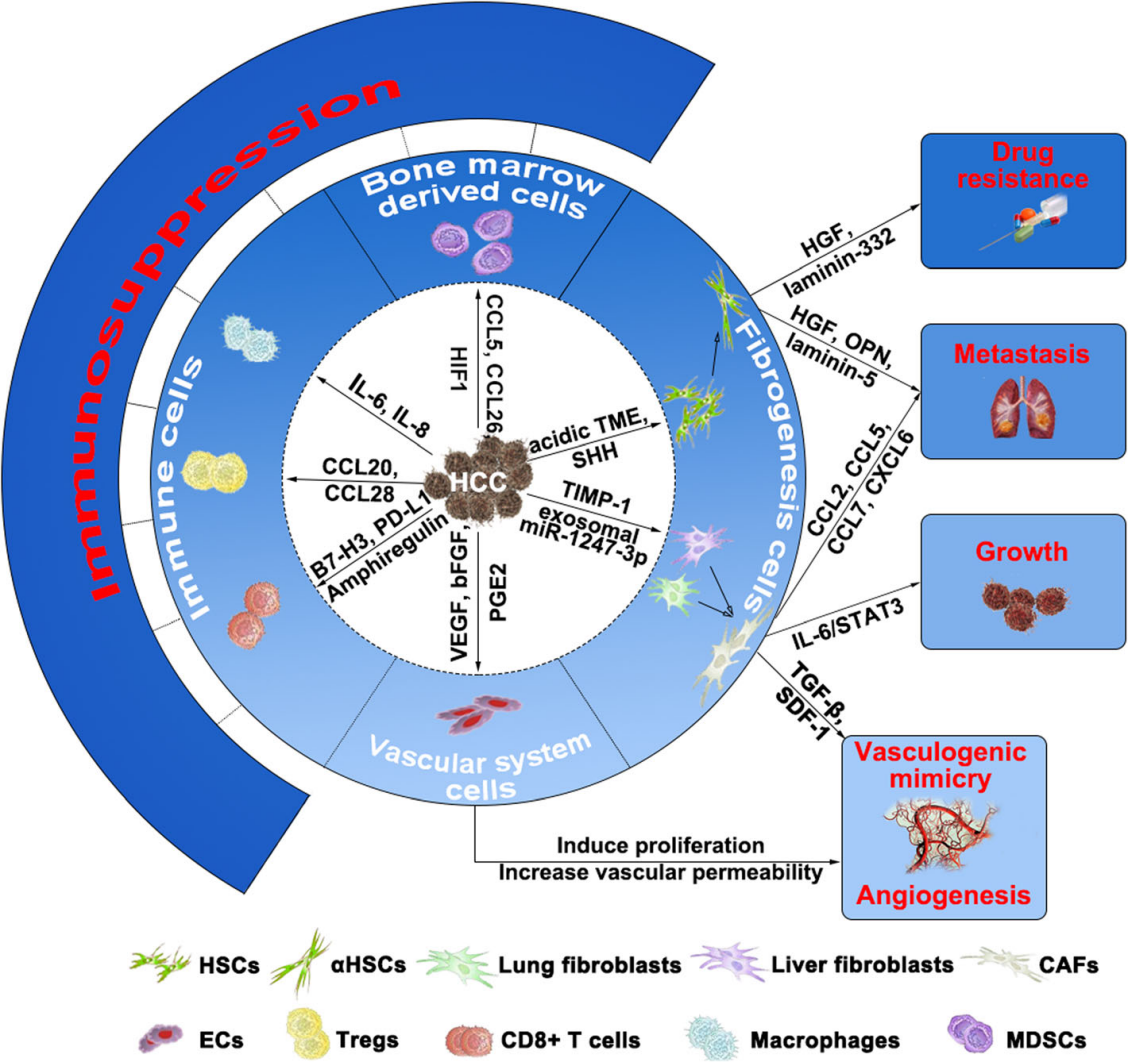
Complex interactions between HCC cells and stromal cells influence HCC progression. The main types of stromal cells in the HCC microenvironment are fibrogenesis cells (HSCs, fibroblasts), vascular system cells (ECs), immune cells (CD8^+^ T cells, Tregs, macrophages) and bone marrow-derived cells (MDSCs). HCC cells can “educate” these cells by different mechanisms. They can activate HSCs through secreting SHH and creating an acidic HCC microenvironment, and the latter can promote HCC drug resistance and metastasis by HGF, OPN, laminin-5 and laminin-332. HCC cells activate the conversion of liver and lung fibroblasts to CAFs by secreting TIMP-1 and exosomal miR-1247-3p, respectively, thus CAFs promote HCC growth through the IL-6/STAT3 pathway and secrete CCL2, CCL5, CCL7, CXCL6, TGF-β and SDF1 to facilitate HCC metastasis and vasculogenic mimicry. Hepatoma cells recruit MDSCs, Tregs and macrophages by secreting CCL5, CCL26, HIF-1, CCL28, CCL20, IL-6 and IL-8 and inhibit CD8^+^ T cells through the upregulated expression of amphiregulin, B7-H3 and PD-L1 to inhibit antitumor immunity. They can also promote EC proliferation to enhance angiogenesis

Tumors are composed of tumor cells and tumor stroma. The stroma involves different cellular and noncellular elements and is termed the tumor microenvironment (TME). The TME consists of stromal cells such as tumor-associated fibroblasts (TAFs), tumor endothelial cells (TECs), immune and inflammatory cells, bone marrow-derived cells, and noncellular elements, such as the extracellular matrix (ECM) and diffusible cytokines, chemokines or enzymes that establish a complex cross-talk with the tumor [10]. Interactions between tumor cells and the TME greatly affect tumor initiation, progression and drug resistance and may become a new target for tumor therapy [11]. The main types of stromal cells in the HCC microenvironment are hepatic stellate cells (HSCs), fibroblasts, endothelial cells (ECs), adipocytes, and immune and inflammatory cells – including CD8^+^ T cells, regulatory T cells (Tregs), macrophages, dendritic cells (DCs) and myeloid-derived suppressor cells (MDSCs) [12–15], and their complex interactions with HCC create a microenvironment suitable for tumor progression (Fig. 1). In the healthy liver, HSCs are in a quiescent state and can be activated by liver damage. Activated HSCs (aHSCs) acquire a myofibroblast phenotype characterized by upregulated expression of alpha- smooth muscle actin (α-SMA) and increased production of ECM components, cytokines and growth factors [12]. HSCs can be activated by an acidic HCC microenvironment and soluble factors secreted by HCC cells, such as sonic Hh (SHH), and influence HCC growth, metastasis, angiogenesis, drug resistance and immunosuppression [16, 17]. Activated HSCs can secrete hepatocyte growth factor (HGF), osteopontin (OPN), and laminin-5 and promote HCC metastasis [16, 18, 19]; they can also lead to drug resistance by producing HGF and laminin-332 [20, 21] and inhibiting the activation of p53 [22]. Active angiogenesis is a hallmark of malignant tumors, and aHSCs can induce HCC angiogenesis through several angiogenic factors, such as vascular endothelial growth factor (VEGF), angiogenin1 (ANG1) and interleukin-8 (IL-8) [17, 23]. In addition, aHSCs can significantly increase MDSCs and Tregs and induce cytotoxic T-cell apoptosis in the HCC microenvironment [24–26]. Fibroblasts in cancer tissues are also known as cancer-associated fibroblasts (CAFs) and represent a major component of the stromal cells that surround cancer cells, especially in HCC, which mainly occurs in fibrotic or cirrhotic livers [27]. Researchers have shown that HCC cells activate liver fibroblast conversion to CAFs by secreting tissue inhibitor of metalloproteinase-1 (TIMP-1), and the latter can promote HCC growth through the IL-6/STAT3 pathway [28]. In addition to the activation of liver fibroblasts, HCC-derived exosomal miR-1247-3p can induce lung fibroblast activation into CAFs to create a pre-metastatic niche suitable for lung metastasis [29]. HCC-associated fibroblasts can also secrete chemokines, such as CCL2, CCL5, CCL7 and CXCL6, to facilitate HCC metastasis through Hh and TGF-β signaling [30]. Vasculogenic mimicry (VM) is a special pattern of blood supply for malignant tumors. It is a kind of vascular-like structure formed by aggressive tumor cells through self-deformation and extracellular matrix remodeling [31]. CAFs in the HCC microenvironment have been shown to promote VM by paracrine transforming growth factor-beta (TGF-β) and stromal cell-derived factor 1 (SDF1) [32]. CAFs also have a critical role in immunomodulation. They can recruit DCs through an SDF-1α-dependent mechanism and transform normal DCs into immunosuppressive DCs by secreting interleukin-6 (IL-6) and inducing STAT3 activation and indoleamine 2,3-dioxygenase (IDO) secretion [33]. HCC is characterized by hypervascularity, and angiogenesis is considered indispensable for tumor growth. ECs in the TME, which are also known as TECs, have phenotypic and functional characteristics different from those of normal ECs. TECs in HCC bear increased angiogenic surface receptors, such as vascular endothelial growth factor receptor (VEGFR), epidermal growth factor receptor (EGFR), platelet-derived growth factor receptor (PDGFR) and CXCR and have increased permeability [12]. HCCs can secrete vascular endothelial growth factor (VEGF), basic fibroblast growth factor (bFGF) and phenyl glycidyl ether 2 (PGE2) to induce EC proliferation [34–37] and excrete exosomal microRNA-103 to increase vascular permeability and promote metastasis [38]. Adipocytes are an important component of the HCC microenvironment in patients with nonalcoholic fatty liver disease. HCC cell-derived exosomes can be actively internalized by adipocytes and cause significant transcriptomic alterations that induce an inflammatory phenotype in adipocytes (upregulated expression of IL-6, IL-8 and monocyte chemoattractant protein1 (MCP1)). Subsequently, they promote HCC growth, enhance angiogenesis, and recruit more macrophages to the HCC microenvironment [15]. The immune response in the tumor and TME is an important regulator of progression in many cancers. Hepatoma cells recruit MDSCs and Tregs by secreting chemokines, such as CCL5, CCL26, hypoxia inducible factor1 (HIF-1), CCL28 and CCL20, and inhibit antitumor immunity [39–43]. They can also activate immunosuppressive Tregs and inhibit CD8^+^ T cells via upregulated expression of amphiregulin, B7-H3 and programmed death ligand-1 (PD-L1) [44, 45]. Macrophages in the TME are termed tumor-associated macrophages (TAMs), and they can be recruited into the HCC microenvironment and polarize into their M2 phenotype by stimulation with inflammatory chemokines, such as IL-6 and IL-8, and thus promote HCC metastasis [46, 47].

Multipotent mesenchymal stromal cells (MSCs) are fibroblast-like plastic-adherent cells that have a wide range of tissue sources. They were first isolated from bone marrow [48–50] and subsequently from other tissues, such as adipose tissue [51], umbilical vein tissue [52], umbilical cord blood [53], fetal liver [54], synovium [55, 56], amniotic fluid [57], placenta [58, 59], Wharton’s jelly [60], human umbilical cord perivascular tissue [61], human periodontal ligament tissue [62] and dental pulp [63]. MSCs have self-renewal [64, 65] and multipotent differentiation capacities; these cells can proliferate rapidly and differentiate into mesodermal cells, such as osteoblasts, adipocytes, and chondrocytes [50], as well as neurons [66], cardiomyocytes [67], endothelial cells [68], pancreatic islet beta-cells [69], retinal cells [70] and other cells under the appropriate conditions. Because this cell population has been shown to possess the capacity for self-renewal and differentiation, characteristics that are typically associated with stem cells, many investigators refer to these cells as mesenchymal stem cells [71]. The International Society for Cellular Therapy (ISCT) has suggested “multipotent mesenchymal stromal cells” as the standard designation [72], which satisfies the minimal identification criteria, as follows: anchorage dependence; expression of the surface molecules CD105, CD73 and CD90; lack of expression of CD45, CD34, CD14 or CD11b, CD79α or CD19 and HLA-DR; and differentiation into osteoblasts, adipocytes, or chondroblasts in vitro [73]. In recent decades, researchers have successfully isolated MSCs from many types of tumor tissue, such as gastric cancer [74], breast cancer [75], ovarian cancer [76], prostate cancer [77], HCC [78], colon cancer [79], glioma [80] and pancreatic cancer [81] and suggested that MSCs are a special type of stromal cell in the tumor microenvironment that affects tumor development (Table 1). Many studies have shown that MSCs can migrate to wounded microenvironments and tumor sites as special stromal cells and participate in injury repair and tumor development in vitro and in vivo. Such properties have made MSCs ideal carriers for tumor-targeted therapies [82–87]. In this review, we describe and discuss the phenomena and mechanisms by which MSCs migrate to the liver fibrosis microenvironment and participate in HCC initiation, along with their recruitment to the HCC microenvironment and their dual tumor promotion and inhibition role in HCC progression; in addition, we discuss the direct evidence demonstrating that MSCs are present in clinical HCC tissue specimens. We will also discuss their widespread applications in HCC-targeted therapy. Therefore, we hope to clarify the critical roles of MSCs in HCC initiation, progression and therapy.

**Table 1:** Mesenchymal stromal cells –special type of stromal cells in tumor microenvironment

Tumor type	Isolation technique	Morphology	Surface markers	Multilineage differentiation	Phenotype and Function	Reference
Gastric cancer	Tissue pieces (1-3 mm^3^) floating culture for 15 days	Long, spindle-shaped fibroblasts	Positive: CD13, CD29, CD44, CD105, HLA-I Negative: CD34, CD38, CD133, CD31, HLA-DR	Adipogenic differentiation (Oil Red O stain) Osteogenic differentiation (ALP stain)	–	Cao et al. [74]
Breast cancer	Monolayer Culture of single-cell suspension isolated from 0.1% type I collagenase-treated tumor tissues	Fibroblastic morphology	Positive: CD90, CD29, CD105, CD73, CD166 Negative: CD31, CD144, CD14, CD45, HLA-DR	Adipogenic differentiation (Oil Red O stain) Osteogenic differentiation (ALP and von Kossa stain) Chondrogenic differentiation (toluidine blue, Alcian blue, Safranin O and HE staining)	Phenotype: Myofibroblast Function: In vitro (enhance mammosphere formation) In vivo (promote growth)	Yan et al. [75]
Ovarian cancer	Monolayer Culture of single-cell suspension isolated from mechanically dissected and filtered tumor tissues or tissue pieces (4*4*2 mm) adherent culture	Fibroblastic morphology	Positive: CD105, CD73, CD90, CD44 Negative: CD14, CD45, CD34, CD133	Adipogenic differentiation (Oil Red O stain) Osteogenic differentiation (Alizarin Red S stain) Chondrogenic differentiation (Alcian Blue stain)	Function: In vitro (promote tumor cell stemness) In vivo (promote growth)	Mclean et al. [76]
Prostate Cancer	Monolayer Culture of single-cell suspension	Fibroblast-like	Positive: CD90, CD105, CD73 Negative: CD45, CD34, CD11b, CD19, HLA-DR	Adipogenic differentiation (Oil Red O stain) Osteogenic differentiation (Alizarin Red S stain) Chondrogenic differentiation (Safranin O stain)	Phenotype: Myofibroblast Function: In vivo (traffic to prostate cancer xenografts)	Brennen et al. [77]
HCC	Monolayer culture of a single-cell suspension isolated from 0.1% type I collagenase-treated tumor tissues	Fibroblastic morphology	Positive: CD29, CD73, CD166, CD90, CD105 Negative: CD45, CD14, CD144, CD31	Adipogenic differentiation (Oil Red O stain) Osteogenic differentiation (ALP and von Kossa stain)	Phenotype: Myofibroblast Function: In vitro (promote proliferation, tumor sphere formation, migration) In vivo (promote growth, metastasis)	Yan et al. [78]
Colon cancer	Monolayer culture of a single-cell suspension isolated from collagenase-digested tumor tissues for 12 days	Fibroblast-like morphology	Positive: CD166, CD13, CD44, CD14, Negative: CD133 CD45, CD34, CD31	Adipogenic differentiation (Oil Red O stain) Osteogenic differentiation (ALP stain)	Function: In vitro (promote proliferation, migration, invasion, tumor sphere formation) In vivo (promote growth, metastasis)	Lin et al. [79]
Glioma	Monolayer culture of a single-cell suspension isolated from mechanically dissected and filtered tumor tissues	Spindle-shaped morphology	Positive: CD105, CD73, CD90, Negative: CD45, CD34	Adipogenic differentiation (Oil Red O stain) Osteogenic differentiation (Alizarin Red S stain) Chondrogenic differentiation (Alcian Blue stain)	Function: In vitro (promote proliferation) In vivo (promote growth)	Hossain et al. [80]
Pancreatic cancer	Culture outgrowth method	Fibroblast-like morphology	Positive: CD90, CD49α, CD44, CD73	Adipogenic differentiation (Oil Red O stain) Osteogenic differentiation (Alizarin Red S stain) Chondrogenic differentiation (Alcian Blue stain)	Phenotype: Myofibroblast Function: In vitro (promote proliferation, invasion) In vivo (promote growth, metastasis)	Waghray et al. [81]

### MSCs migrate to the liver fibrosis microenvironment and are involved in HCC initiation

Throughout their genesis and development, most HCCs undergo a long process that starts with chronic liver disease and liver damage, mainly due to chronic hepatitis B virus (HBV) infection, alcoholic liver disease, and nonalcoholic steatohepatitis (NASH). These harmful factors cause hepatocellular necrosis, apoptosis or dysfunction and cause infiltration of immune inflammatory cells. Injury and the inflammatory microenvironment stimulate HSC activation, eventually leading to liver fibrosis. The process of liver fibrosis may promote reduplicative proliferation, regeneration and repair of hepatocytes and make hepatocytes prone to spontaneous mutations, leading to progression to HCC. Chronic HBV infection accounts for the majority of liver fibrosis and HCC cases, especially in most Asian countries [88]. Long-term and repeated viral infection-induced liver inflammation caused by host immune responses leads to hepatocyte necrosis, HSC activation and subsequent liver fibrosis. Alcoholic hepatitis is a type of inflammation of the liver due to alcohol abuse that is also characterized by hepatocyte necrosis and infiltration by inflammatory cells, such as neutrophils. With the globalization of obesity and its related metabolic syndrome, NASH has become an important cause of chronic liver disease in developed countries such as Europe and America. Hepatocyte apoptosis in NASH patients induced by oxidative stress, endoplasmic reticulum stress, and autophagy stimulates HSC activation and liver fibrosis progression through the production of chemokines and cytokines [89]. The progression of liver fibrosis caused by different chronic damage factors creates a chronic injury and inflammatory fibrotic microenvironment, which can recruit MSCs to participate in liver injury repair [90]. During this process, MSCs incorporated into the fibrotic liver may be involved in HCC initiation..

### MSCs migrate to the liver fibrosis microenvironment

**Fig. 2:**
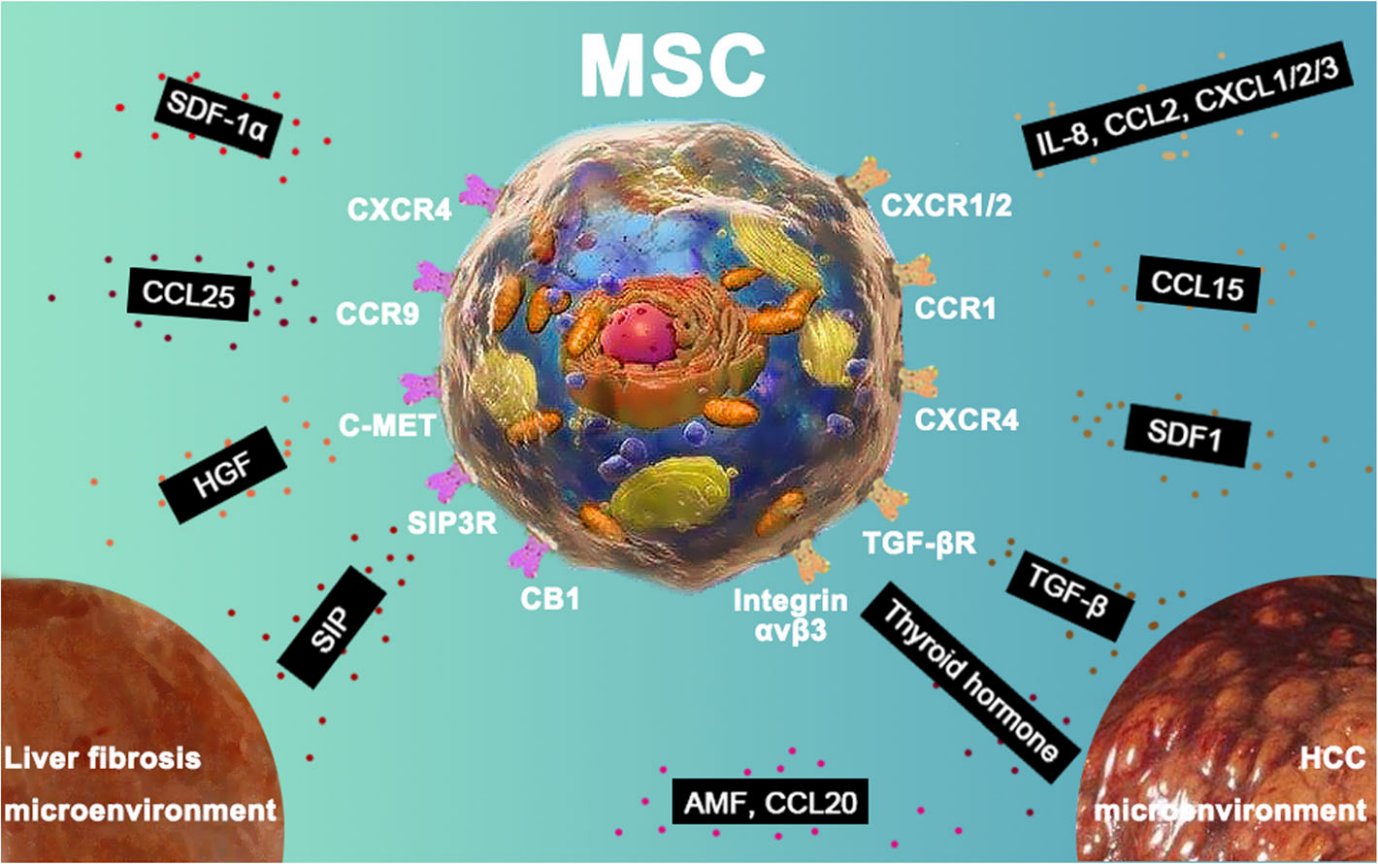
Chemotaxis mechanisms that mediate MSC migration to the liver fibrosis and HCC microenvironment. MSCs can migrate to the liver fibrosis and HCC microenvironment, and this capacity has made MSCs ideal carriers for targeted therapies. MSCs migration to the liver fibrosis microenvironment can be mediated by the chemokine SDF-1α/CXCR4 and CCL25/CCR9 axes and the growth factor HGF via activation of c-MET. In addition to the chemokines and inflammatory growth factors known to exert potent cellular chemotactic effects, the sphingolipid metabolite S1P is one of the most important candidates for the induction of MSC mobilization via SIP3R. CB1 can also mediate homing of MSCs triggered by chronic liver injury. MSCs can be recruited into the HCC microenvironment by AMF and several chemokines, including IL-8, CCL2, CXCL1/2/3, CCL20 and CCL15/CCR, and SDF-1/CXCR4. TGF-β/TGF-βR are also involved in this process. Thyroid hormones can increase hMSC migration to HCC stroma via integrin αvβ3

In 1970, Friedenstein et al. first described cells derived from monolayer cultures of guinea-pig bone marrow and spleen cells as “fibroblast colony-forming units” (CFU-F) with fibroblast morphological features, high proliferative activity, and spontaneous and induced osteogenic differentiation [48]. These cells were later determined to be multipotent MSCs [72]. Over the next three decades, many preclinical and clinical studies have revealed that these cells rapidly respond to damage “signals” and migrate toward wounded microenvironments, such as injured spinal cord tissue [82] and wounded skin [83], and participate in wound healing or tissue regeneration. The signals mediating MSC migration mainly include inflammatory growth factors, such as platelet-derived growth factor (PDGF), insulin growth factor (IGF), HGF, fibroblast growth factor (FGF), and TGF-β [90–93], and chemokines, including SDF-1/CXCL12 [94, 95], CCL25 [96], CXCL10 and CXCL11 [97], CXCL8 [98], interleukin-1 (IL-1) [99], IL-6 [100], complement component 1 subcomponent q (C1q) [101], C3a and C5a [102], and tumor necrosis factor-alpha (TNF-α) [103]. Chronic liver injury, such as that induced by hepatitis B infection, generates a chronic inflammatory and fibrotic microenvironment and recruits MSCs to participate in the repair of liver damage and progression of fibrosis. Researchers have documented that MSC migration to the liver fibrosis microenvironment can be mediated by sphingosine 1-phosphate (S1P), SDF-1α, CCL25 and HGF (Fig. 2). Liu et al. indicated that BMSCs integrated into a liver fibrosis mouse model induced by CCl_4_ and promoted liver fibrogenesis, and SDF-1α and CXCR4 were found to be the key chemotactic axis regulating MSC migration from the bone marrow to the liver [104]. Chen et al. reported that the SDF-1α/CXCR4, CCL25/CCR9 and HGF/c-MET (mesenchymal-epithelial transition factor) axes were responsible for BMSC migration [105]. In addition to the chemokines and inflammatory cytokines known to exert potent cellular chemotactic effects, the sphingolipid metabolite S1P is one of the most important candidates for cell mobilization induction. Li et al. reported that the concentration gradient of S1P between the bone marrow and damaged liver induced by CCl_4_ induced BMSC migration via the S1P3 receptor and BMSCs that migrated to the liver injury microenvironment differentiated into myofibroblasts, which play a central role in the pathogenesis of liver fibrosis [106]. In addition to the receptors mentioned above, cannabinoid receptor 1 (CB1) can also mediate the homing of BMSCs triggered by chronic liver injury [107]. Although many preclinical and clinical studies have focused on the applications of MSCs in the treatment of liver fibrosis and cirrhosis, clarifying the chemotaxis mechanisms will greatly improve the therapeutic effect.

### MSCs and hepatocarcinogenesis

The malignant transformation of MSCs has been reported in many studies. The underlying molecular mechanisms involved in this process remain unclear. Houghton et al. first found that gastric cancer (a type of epithelial tumor) can originate from the malignant transformation of bone marrow-derived cells (BMDCs). They indicated that BMDCs can home to the chronic gastric inflammatory microenvironment caused by chronic *Helicobacter* infection and participate in the repair of damaged gastric mucosa. Hyperactive proliferation of BMDCs increases the possibility of mutation and eventual progression to gastric cancer [108]. Tso et al. found that primary glioblastoma tumors and their passaged tumor cell lines expressed the cellular and molecular characteristics of MSCs. Further, when treated with adipogenic, osteogenic, or chondrogenic induction medium, primary glioblastoma cell lines could differentiate into mesenchymal lineage cell types [109]. The malignant transformation phenomenon of MSCs in Ewing’s sarcoma is similar to that of these tumors [110]. This evidence suggests that MSCs may be the third possible cellular origin of cancer, paralleling the maturation arrest of tissue stem cells and dedifferentiation of mature cells.

As is well known, the Myc gene family, including c-Myc, N-Myc, and L-Myc, is a group of genes that play critical roles in promoting cell proliferation, immortalization, differentiation, dedifferentiation and transformation; for instance, they can control the differentiation of adipose stem cells and regulate adipogenesis [111]. Most importantly, Myc, and especially c-Myc, has been regarded as one of the most critical oncogenes that participate in carcinogenesis [112]. Research first described the role of MSCs in hepatocarcinogenesis in 2007. Studies have indicated that MSCs derived from bone marrow in rats transfected with the K-ras oncogene alone, or with c-myc and K-ras combined, differentiated into HCC cells in vivo and resulted in hepatocarcinogenesis after portal vein injection [113]. Because of their potency to differentiate into hepatocytes, MSCs were thought to have great potential for liver regeneration [114], and it was reported that MSCs have in vivo hepatic differentiation potential and a therapeutic effect on liver fibrosis [115]. However, when these cells differentiate into hepatocytes, abnormal expression or localization of certain genes may be associated with a tumoral phenotype, such as the abnormal nuclear translocation of β-catenin [116]. In 2014, more direct evidence suggested that MSCs may initiate HCC. Researchers demonstrated that the HCC cell line SK-Hep-1 expressed most classical cell surface markers of human MSCs, such as CD73, CD90, CD105, CD44, CD29, CD146 and CD166, but expressed no hematopoietic markers or endothelial markers. When treated with osteogenic and adipogenic differentiation medium, these cells differentiated into osteogenic cells and adipogenic cells. Most importantly, SK-Hep-1 cells represented steady self-renewal and tumorigenic and metastatic capacity, consistent with cancer stem cells [117]. Although these studies indicated that MSCs may be involved in hepatocarcinogenesis, more definitive evidence is needed to identify the malignant transformation of MSCs in vivo and elucidate its causative mechanism.

### MSCs migrate to the HCC microenvironment and are involved in HCC progression

Tumors can be considered “wounds that never heal” and are sites of inflammatory cytokine and chemokine production [118–121], and most HCC cases are caused by chronic liver diseases with varying degrees of chronic inflammatory fibrosis, which may partially enable MSCs to home to and participate in HCC progression.

#### MSCs migrate to the HCC microenvironment

Studeny et al. first showed that human bone marrow-derived MSCs preferentially incorporate into melanomas in the lungs rather than in the lung parenchyma and in subcutaneous melanomas rather than in other normal organs, such as the liver, after intravenous injection. These MSCs can effectively secrete engineered interferon-β (IFN-β) locally to inhibit tumor growth [84]. This research has led scientists to focus on the characteristics of the directional migration of MSCs to tumor sites and the application value in tumor-targeted therapy. Until 2008, many studies described “tumor tropism” and “targeted delivery” of multipotent MSCs, including breast carcinoma [122], glioma [85], ovarian carcinoma [123], Kaposi’s sarcoma [124], lung cancer [125], and colon cancer [126]. Monitoring MSC tropism for tumors and wounded microenvironments by directly labeling cells with luciferase for in vivo bioluminescent imaging was first reported in 2009. Previously, MSC dispersion in recipients was monitored by immunohistochemical staining or fluorescent visualization after the animals were sacrificed. However, in vivo imaging allows for long-term dynamic monitoring of MSC distribution and variation in vivo [86].

Multipotent MSC migration to HCC has been reported in many studies using in vitro assays and animal models, and no relevant clinical trials have demonstrated this characteristic. This migration was initially reported in 2008. Researchers found that interleukin-12 (IL-12) gene-engineered murine MSCs were preferentially present in primary tumor sites and spontaneous metastatic sites pre-established by subcutaneously injecting Hca hepatoma cells, representing tumor inhibition [127]. Subsequently, Garcia et al. analyzed the capacity of human bone marrow-derived MSCs to migrate or anchor to HCC and its fibrotic microenvironment in vitro and in vivo [128]. In vitro assays showed that human MSCs (hMSCs) migrated through polycarbonate filters and adherently invaded through type IV collagen and an endothelial cell layer previously incubated in polycarbonate filters. This process occurred in response to cell-conditioned media (CCM) generated from HCC cell lines (Hep3B, Huh7, PLC/PRF/5), a hepatic stellate cell line (LX-2), and tumor-conditioned media (TCM) collected from primary cultures of fresh tumor tissues from an HCC patient (HC-PT-5) or subcutaneous tumors induced by injecting HC-PT-5 or Huh7 cells into nude mice. In vivo fluorescence imaging of subcutaneous and orthotopic hepatocellular carcinoma models with or without fibrosis showed that hMSCs were distributed in tumors and fibrotic microenvironments, indicating that liver cancer and its fibrotic microenvironment efficiently recruit MSCs. This research demonstrated that HCC cells and HSCs induce MSC migration to HCC and the fibrotic microenvironment by secreting soluble molecules. Because most HCC patients exhibit varying degrees of liver cirrhosis and many preclinical and clinical studies have demonstrated that MSCs recruited to the liver can effectively treat liver fibrosis, determining the critical molecules in this process will also be beneficial for HCC-targeted therapy.

Garcia et al. suggested that the autocrine motility factor (AMF) secreted by HCC cells enhanced the recruitment of human MSCs derived from bone marrow, adipose tissue and umbilical cord perivascular cells, and HCC tropism of human umbilical cord perivascular cells was increased more than bone marrow MSCs [129, 130]. In addition to AMF, Garcia et al. found that the IL-8, CXCL1, CXCL2, CXCL3 and CCL2 are also important for in vitro MSC migration towards human HCC via binding with CXCR1/2 expressed on the MSC surface. In addition to inducing MSC migration towards HCC cells, HCC-released factors can enhance the migration capability of MSCs after exposure to HCC-conditioned media (CM), which suggests that HCC cells can “educate” MSCs, as discussed below [131]. Factors responsible for multipotent MSCs homing to liver cancers also include chemokines such as CCL15 [132], CCL20 [133], and SDF-1α [134]. Better clarification of the molecular signals that recruit MSCs to HCC may permit the efficient targeted delivery of MSCs for therapeutic purposes. Investigators have collected CM generated from HCC cell lines (MHCC-97H, HepG2, Huh-7) and analyzed these cytokine profiles compared with CM collected from a human immortalized liver cell line (LO2). The chemokine CCL15 was the most abundant in all three HCC cell lines. An in vitro transwell migration assay suggested that CCL15 may be involved in human MSC chemotaxis towards HCC, and this chemotactic effect by CCL15 was mediated via CCR1 on hMSCs. An orthotopic transplantation tumor model of HCC in nude mice established by 97H-CCL15-shRNA cells attracted fewer systematically delivered human bone marrow-derived MSCs, further demonstrating the induced migration effect of CCL15 [132]. A similar study showed that only CM generated from the Huh-7 cell line attracted human bone marrow-derived MSCs in an in vitro transwell migration assay rather than the HepG2 cell line. The results of a human cytokine antibody array differed from the results of the abovementioned study, although either chemokine CCL15 levels were increased or MSC migration was enhanced [133]. The differences between these two studies may have been due to the distinct experimental conditions, such as the method used to generate the CCM, which indicates that culture conditions, including culture media with or without serum and culture time, must be reported. This finding also suggested that it is important to note individual differences among HCC patients when conducting clinical trials on MSCs. Mardomi et al. also studied the HCC tropism of hMSCs and demonstrated that CXCR4/CXCL12 and TGF-β/TGF-βR may be involved in this process [134]. Interestingly, thyroid hormones can also increase hMSC migration to HCC stroma via integrin αvβ3 [135] (Fig. 2).

It is worth noting that although many soluble molecules secreted by hepatoma cells can induce MSC migration toward HCC, the ability of various HCC cell lines to induce MSC migration is quite different. Clarifying the intrinsic differences between HCC cells, such as differential expression of membrane proteins and activation of different signaling pathways, can further elucidate the mechanisms of chemotaxis. HCC is a heterogeneous cell population, with highly malignant cancer stem cells (CSCs) presenting steady self-renewal, tumorigenic and metastatic capacity. Epithelial cell adhesion molecule (EpCAM) is a known surface marker of liver cancer stem cells (LCSCs) and a prognostic marker of HCC. It not only can mediate intercellular adhesion but also influences cell signaling after being activated [136]. Endaya et al. found that HCC-bearing mice with high activation of EpCAM signaling (characterized by EpCAM cleavage followed by the intracellular domain of EpCAM (EpICD) entering into the cell nucleus and transcription of downstream target genes-c-Myc) can recruit more human bone marrow-derived MSCs [137]. This research documented that highly oncogenic HCC cells can induce increased MSC migration and enable MSCs to be an effective carrier for HCC-targeted therapy. Although this study was pioneering, the authors did not identify the specific chemoattractants responsible for MSC migration. The recruitment of MSCs towards EpICD-over-expressing HCC was mediated by CM. Further research further determine the relevant molecules in the CM and connect these molecules to EpCAM signaling. While the phenomenon of multipotent MSCs homing to tumors has been extensively documented, further clarification of the tropism mechanism is needed.

#### MSCs are involved in HCC progression

Since MSCs can be recruited to the tumor microenvironment, what is their effect on tumor progression? In 2003, investigators found that the murine C3H10T1/2 MSC line and human bone marrow-derived MSCs favor B16 melanoma growth in allogeneic mice due to immunosuppression [138]. In contrast, Khakoo et al. indicated that human bone marrow-derived MSCs homed to Kaposi’s sarcoma after intravenous injection, dose-dependently inhibiting KS tumor growth by inhibiting Akt activation [124]. These two contrasting studies suggested that the role of MSCs in the TME may depend on the tumor type and immune state. MSCs may promote tumor growth by hindering antitumor immunity in the body with normal immune function and inhibit tumor progression in a tumor immunosuppression microenvironment.

Multipotent MSCs also play a dual role in HCC progression (Table 2.). Many studies have shown that MSCs induce apoptosis, inhibit HCC cell proliferation, migration and invasion in vitro, and suppress tumor growth and metastasis in vivo. Qiao et al. showed that CM generated from Z3 cells (human MSCs established from fetal dermal tissue) and BMMS-03 cells (human MSCs derived from fetal bone marrow) suppressed H7402/HepG2 human hepatoma cell proliferation and that NF-κB downregulation may be involved in this suppression [139]. In addition to NF-κB downregulation, Notch1 signaling is reported to be involved in inhibiting proliferation [140]. In another study from the same year, Qiao et al. also indicated that H7402/HepG2 human hepatoma cells subcutaneously coinjected with Z3 cells in severe combined immunodeficiency (SCID) mice delayed tumor formation time and inhibited hepatoma growth, which may be mediated by the Wnt signaling pathway [141]. In addition to bone marrow-derived MSCs, researchers also found that CM collected from human adipose-derived MSCs decreased proliferation and induced apoptosis in human hepatoma cells in vitro. Furthermore, human fetal MSCs suppressed HCC growth in SCID mice [142, 143]. Notably, except for soluble factors in the CM or tumor microenvironment, extracellular vesicles, such as exosomes and microvesicles released from MSCs, also inhibit HCC. Bruno et al. found that microvesicles (MVs) derived from human bone marrow MSCs can incorporate into HepG2 cells and significantly inhibit proliferation and induce apoptosis in vitro. The molecular changes in the gene array profiles of MV-treated HepG2 cells mainly related to cell cycle arrest and may explain the inhibitory effects of MVs. To further demonstrate the inhibitory effect of MVs in vivo, researchers established tumor-bearing mice generated by subcutaneously injecting HepG2 cells in SCID mice and found that tumor growth was significantly inhibited after MV injection [144]. Similarly, Ko et al. also established tumor-bearing rats generated by injecting rat N1S1 cells into the subcapsular site of the left lobe and administered ADMSC-derived exosomes via the penile vein to observe the effect of exosomes on HCC growth. The results showed that ADMSC-derived exosome-treated HCC-bearing rats presented reduced tumor volume, lower-grade HCC and significantly higher percentages of circulating and intratumoral NKT-cells [145]. Li et al. showed that MHCC97-H human HCC cells presented reduced invasion potential in an in vitro invasion assay after treatment with CM generated from human bone marrow-derived MSCs, and these cells suppressed tumor metastasis in subcutaneous and orthotopic hepatoma models in nude mice after intravenous injection. The efficient inhibition of invasion and metastasis possibly occurred via downregulated TGF-β1 expression in hepatoma cells and upregulated stromal differentiation in MSCs [146, 147].

**Table 2:** Mesenchymal stromal cells inhibit or promote HCC progression

Dual function	Impact on biological behavior	MSCs	HCC cell line	Molecule mechanism	Reference
Inhibition	Inhibit proliferation	Z3 and BMMS-03	H7402/HepG2	NF-κB signaling	Qiao et al. [139]
		MSCs	HepG2	Notch1 signaling	Abdel Aziz et al. [140]
		fMSCs	Huh7	IGF-1R/PI3K/Akt signaling	Yulyana et al. [143]
		AMSCs	HepG2, Huh7, SMMC7721, Bel7402	Akt signaling	Zhao et al. [142]
	Induce apoptosis	AMSCs	,SMMC7721	Akt signaling	Zhao et al. [142]
		BMSCs	HepG2	Microvesicles	Bruno et al. [144]
	Inhibit growth	AMSCs	N1S1 rat HCC cells	Exosomes promote NKT-cell antitumor responses	Ko et al. [145]
	Inhibit invasion and metastasis	BMSCs	MHCC97-H	TGF-β signaling	Li et al. [146]
Promotion	Promote EMT	MSCs	SK-Hep-1	OPN	Bhattacharya et al. [151]
	Promote proliferation	BMSCs	HepG-2	–	Gong et al. [152]
	Promote angiogenesis	BMSCs	HepG-2	–	Gong et al. [152]
	Promote invasion and metastasis	BMSCs	SNU-398	CXCR4	Fontanella et al. [153]
		UCMSCs	HCCLM3	TGF-β signaling	Liu et al. [154]

Conversely, increasing evidence suggests that MSCs may promote HCC progression. Epithelial-mesenchymal transition (EMT) of cancer cells plays an important role in HCC progression. EMT contributes to the increased population of cancer stem-like cells (CSCs), which are related to tumor metastasis and chemoresistance [148–150]. Bhattacharya et al. showed that human MSCs differentiated into CAFs, which markedly expressed tenascin-c and SDF-1 and subsequently promoted EMT of the human hepatoma cell line SK-Hep-1 when these two cell types were in an admixture [151]. Gong et al. indicated that HepG-2 cell proliferation was increased when treated with CM generated from adult female bone marrow-derived MSCs, and the latter could promote angiogenesis and enhance the microvascular density in the transplanted hepatoma area in nude mice [152]. Human bone marrow-derived MSCs and umbilical cord MSCs caused increases in hepatoma cell migration and invasion in vitro and in 3D-culture, respectively [153, 154], and this increased invasion may have been due to IL-6 secretion by MSCs [155]. Why MSCs exert the dual roles of tumor promotion and tumor inhibition in HCC is unknown and may depend on various tissue sources, different MSC cell counts and the immune microenvironment in HCC. Understanding the precise effects of this cell type on tumor development, including hepatomas, and clarifying the molecular factors involved may provide new insights into cancer therapy.

#### MSCs exist in HCC tissue specimens

Recognizing tumor tropism and the dual role of MSCs in mouse HCC models raises a new question: what are MSCs in clinical liver cancer tissues, and what role do they play in human HCC? Evidence suggesting that MSCs exist in human solid tumor tissues was first reported in human bone sarcomas. Gibbs et al. found cells derived from human bone sarcomas that bore the MSC markers, stro-1, CD44 and CD105, and these cells could be induced to differentiate along at least two distinct mesenchymal lineages by culturing in osteogenic and adipogenic medium [156]. Subsequently, MSCs were also found in human benign neoplasm-lipoma [157]. Both malignant bone sarcoma and benign lipoma originate from the mesenchyma. Because these two neoplasms, as well as all mesenchymal neoplasms, can arise from the differentiation arrest of MSCs or from MSCs that previously existed in the mesenchymal tissues before tumorigenesis for tissue renewal, it is unsurprising that both bone sarcomas and lipomas contained MSCs.

As mentioned in the previous section, epithelial tumor gastric cancer can originate from bone marrow-derived cells in mouse models with chronic *Helicobacter* infection [108]. Based on this study, Cao et al. initially explored whether MSCs were present in human gastric carcinoma tissue specimens collected surgically and indicated that fibroblast-like cells isolated from human gastric cancer tissues possessed a characteristic MSC morphology, immunophenotype and differentiation potential, all of which were similar to human bone marrow-derived MSCs (hBM-MSCs). In contrast, MSC-like cells in gastric cancer tissues contained more organelles, such as the mitochondria and endoplasmic reticulum, and they proliferated faster than hBM-MSCs [74]. The difference between these two shows the “educational effect” of the tumor microenvironment, which may benefit tumor initiation and progression. This report is also the first to report the existence of MSC-like cells in epithelial tumor tissues. Researchers from the same group then found that MSC-like cells also existed in adjacent noncancerous tissues located more than 5 cm away from the primary gastric cancer sites, with broadly similar morphology, surface markers, stem cell-related gene expression, and differentiation potential but lower proliferation capability, higher migration ability and different microRNA expression profiles than gastric cancer-derived MSCs [158, 159]. The existence of MSCs in other human epithelial tumors was subsequently reported, including in breast [75], ovarian [76], prostate [77], hepatocellular [78, 160, 161], colon [79], glioma [80], and pancreatic cancers [81]. McLean et al. successfully isolated ovarian cancer-associated MSCs from human ovarian cancer tissue specimens. These cells generally had the morphology, surface marker expression, and differentiation potential of adipose and bone marrow-derived MSCs extracted from healthy people. However, the increased expression of acetaldehyde dehydrogenase (ALDH), the capacity to generate single cell clones and the promotion of tumor growth of ovarian cancer-associated MSCs compared with those of bone marrow-derived MSCs suggested an altered phenotype that may favor ovarian cancer progression [76]. In addition to identifying the existence and role of MSCs in the tumor microenvironment, their proportions must be quantified. Brennen et al. collected human prostate cancer specimens, immediately dissociated them into single cell suspensions and quantified the percentage of MSCs by a flow cytometry-based assay prior to expansion in primary culture [77]. Although the number of tumor-associated MSCs is indefinite, as it may depend on tumor type and individual variation, the percentage is likely very small based on current evidence, including 0.3% in human ovarian cancer tissue specimens, 0.01-1.1% in digested prostatectomy tissues and 8.9% of the total CAFs in pancreatic cancer tissues [76, 77, 81].

To date, three papers have discussed HCC-associated MSCs. Yan et al. first showed that MSCs existed in HCC and adjacent tumor-free tissues, which mainly presented a myofibroblast phenotype, and these cells significantly accelerated HCC growth and metastasis in subcutaneous and intrahepatic human HCC nude mouse models, respectively. These authors focused on the mechanisms by which HCC-associated MSCs modulate liver cancer progression along the S100A4-miR155-SOCS1–STAT3-MMP9 axis [78]. Another study from the same year also reported that MSCs were part of the liver cancer microenvironment and promoted tumor progression [160]. The third article was also by Yan et al., who found that HCC-associated MSCs enhanced tumor spheroid formation and CSC marker expression, such as CD90 and CD13, in vitro. These cells also promoted liver cancer stemness, including tumorigenicity and metastasis in vivo, which may be mediated by the interaction between lncRNA-MUF and ANXA2 or miRNA-34a [161]. Notably, CAFs expressing the myofibroblast phenotype is a theoretical concept [162]; thus, the relationship between CAFs and tumor-associated MSCs, as well as their roles in human HCC, remains to be determined.

### MSCs and HCC-targeted therapy

Although many studies using in vitro assays and animal models have suggested that MSCs can migrate to the TME, and this capacity has made MSCs ideal carriers for tumor-targeted therapies, there is little clinical evidence for the recruitment of MSCs to tumor sites. In addition, considering the possibility of malignant transformation and the promotion of tumor progression by MSCs, most studies are still in the preclinical phase, and accumulating evidence now suggests that a novel cell-free therapy, MSC-secreted exosomes, might constitute a compelling alternative because of the advantages over the corresponding MSCs. However, it is worth noting that some clinical trials have begun using MSCs for treatment or adjuvant therapy of certain tumors, including HCC.

#### Preclinical studies of MSCs in HCC-targeted therapy

Scientists have previously focused on the characteristics of the directional migration of MSCs to tumor sites and their application value in tumor-targeted therapy, as researchers found that human bone marrow-derived MSCs genetically engineered with IFN-β preferentially incorporate into melanomas in the lungs rather than the natural lung parenchyma and into subcutaneous melanomas rather than other normal organs, such as the liver, after intravenous injection and locally secrete IFN-β to inhibit melanoma growth [84]. MSCs have since been regarded as ideal targeted-delivery vehicles for tumor-targeted therapy, which could greatly enhance treatment efficacy and reduce adverse effects.

**Fig. 3:**
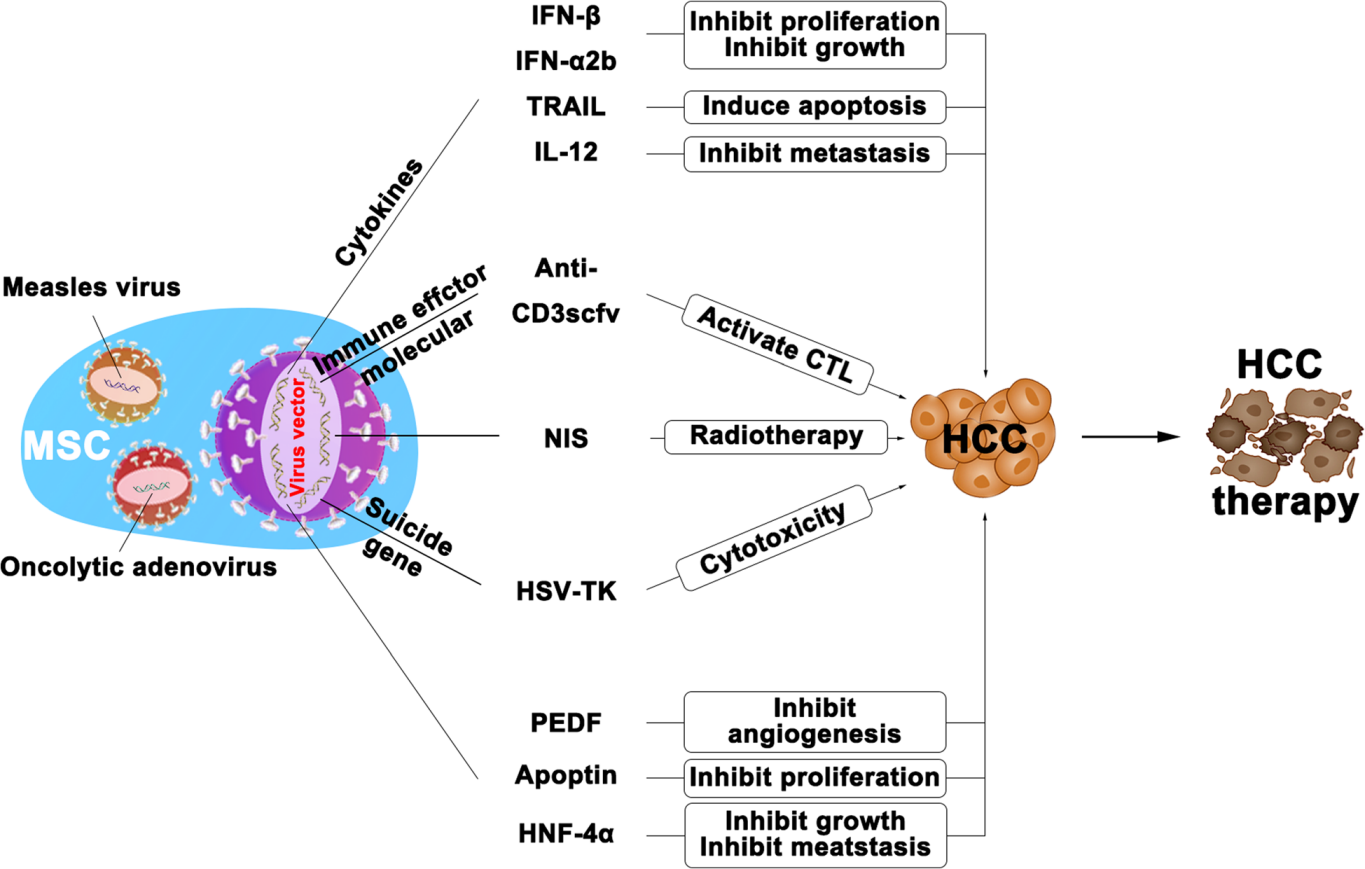
MSC-based HCC-targeted therapies. MSC-based HCC-targeted therapies primarily include genetically engineered MSCs and oncolytic virus-infected MSCs. MSCs engineered with cytokine genes, such as IFN-β, IFN-α2b, TRAIL and IL-12, inhibit HCC proliferation, growth, metastasis and induce apoptosis. MSCs packaging adenovirus expressing anti-CD3scfv can activate CTL and inhibit HCC. Using the NIS gene to modify MSCs provided a novel mechanism for evaluation of MSCs as gene delivery vehicles for tumor therapy and improved the effect of local radiotherapy. MSCs transfected with the suicide gene HSV-TK can transform the prodrug ganciclovir into a cytotoxic drug that kills hepatoma cells. MSCs engineered with PEDF, HNF4α and apoptin genes can inhibit angiogenesis, growth, metastasis and proliferation. Oncolytic viruses used to infect MSCs include measles virus and oncolytic adenovirus, and they presented obvious tumor inhibition in the HCC microenvironment

To the best of our knowledge, preclinical studies on HCC treatment with MSCs primarily include genetically engineered MSCs and oncolytic virus-infected MSCs (Fig. 3). The genes used to modify MSCs to locally treat HCC include cytokines such as IFN-β [127, 163], IFN-α2b [164], tumor necrosis factor-related apoptosis-inducing ligand (TRAIL) [165], IL-12 [166–168]; immune effector molecule anti-CD3scfv [169] and suicide gene HSV-TK (herpes simplex virus-thymidine kinase) [170]. In addition, MSCs engineered with sodium iodide symporter (NIS) [127, 163], pigment epithelial-derived factor (PEDF) [84], hepatocyte nuclear factor 4α (HNF4α) [164] or apoptin [171] genes can also inhibit HCC progression. The oncolytic viruses used to infect MSCs include measles virus [165] and conditionally replicative adenovirus (CRAd) [167, 168], and they presented obvious tumor inhibition. Because human bone marrow-derived MSCs genetically engineered with IFN-β (BMSC/IFN-β) have been applied to treat glioma [172], they were also applied to HCC studies. The results showed that BMSC/IFN-β significantly attenuated HCC HepG2 and Huh7 cell proliferation in vitro by decreasing the proportion of S-phase cells, thus delaying hepatoma formation and inhibiting tumor growth in NOD/SCID mouse models by inhibiting the AKT/FOXO3a pathway [171]. IFN-α is an important cytokine that has been used clinically as a therapeutic strategy for HCC, but its short half-life and systemic toxicity limit its clinical application [173]. MSCs transfected with recombinant human IFN alpha2b (IFN-α2b) overexpression constructs have effectively overcome these limitations [174, 175]. TRAIL (also known as Apo-2 L) is a member of the TNF superfamily. MSCs genetically modified with TRAIL induced hepatoma cell apoptosis alone or in combination with chemotherapeutic agents via death receptor 5 (DR5) [176]. Chen et al. also found that MSCs engineered to secrete IL-12 prevented hepatocarcinogenesis and delayed metastasis without obvious systemic toxic effects [127, 163]. An immunosuppressed microenvironment promotes HCC progression, and MSCs delivering immune effector molecules can activate antitumor immunity. Researchers have indicated that MSCs carrying adenovirus expressing anti-CD3scfv can activate cytotoxic lymphocytes (CTLs) and inhibit HCC [169]. In addition to the abovementioned genes, MSC-based suicide gene therapy also plays a role in HCC-targeted therapy. Niess et al. reported that exogenously added CCL5/HSV-Tk or Tie2/HSV-TK transfected MSCs are recruited to grow HCC xenografts and concomitantly activate the CCL5 or Tie2 promoters within the MSCs. MSCs that mediated introduction of the suicide gene HSV-Tk into tumors, followed by administration of the prodrug ganciclovir, effectively treated experimental HCC [176]. Using the NIS gene to modify MSCs provided a novel mechanism for evaluation of MSCs as gene delivery vehicles for tumor therapy and improved the effect of local radiotherapy [174, 175]. In addition, MSCs engineered with PEDF, HNF4α and apoptin genes can inhibit angiogenesis, growth, metastasis and proliferation [170–172]. MSCs can also serve as vehicles for delivering oncolytic viruses, such as measles virus and oncolytic adenovirus, to eliminate hepatoma cells [177, 178]. Although MSCs engineered to express cytokines, immune effector molecules, suicide genes and others can effectively inhibit HCC progression, these therapies are still a long way from preclinical studies toward clinical application. Considering the potential risk of MSCs in promoting tumor progression in the TME, using MSCs to treat HCC should require strict safety evaluations.

In recent years, accumulating evidence has suggested that a novel cell-free therapy, MSC-secreted exosomes, might constitute a compelling alternative because of its advantages over the corresponding MSCs [179, 180]. Bruno et al. found that microvesicles derived from human bone marrow MSCs inhibited proliferation and induced apoptosis in HepG2 cells, suggesting that unprocessed extracellular vesicles released from MSCs could be a treatment option [145]. In addition, Lou et al. suggested that exporting miR-122 via adipose-derived MSC exosomes can significantly enhance HCC chemosensitivity to sorafenib and may provide a more effective treatment option [181].

### Clinical trials of MSCs in HCC therapy

Registered clinical trials for the treatment of solid tumors with MSCs have primarily focused on ovarian cancer; these clinical trials are still under way, though no results have yet been published. M.D. Anderson Cancer Center sponsored a phase 1 clinical trial to find the highest tolerable dose of human MSCs transfected with interferon beta (MSCs-IFNβ) that can be given to patients with ovarian cancer and to test the safety of MSCs-IFNβ. Mayo Clinic initiated a phase I/II trial to study the side effects and best dose of MSCs infected with oncolytic measles virus encoding NIS (MV-NIS) and to see how well it works in treating patients with ovarian cancer. Although the treatment results have not been published, more attention and patience are needed to promote the clinical transformation of MSCs in tumor therapy.

Although MSCs have been widely used in treating liver cirrhosis in clinical trials, clinical trials for treating liver cancer with MSCs are rare. Only one clinical trial is on the registry and is recruiting subjects. This trial aims to study whether the administration of corticoid hinders or enhances the mobilization of MSCs in the peripheral blood during liver transplantation of HCC patients and whether this effect influences the outcome with respect to graft versus host response. MSCs are a known immune modulator with significant immunosuppressive effects; therefore, if MSCs can be used to decrease the use of immunosuppressive agents in liver transplant patients, there may be a significant decrease in morbidity and mortality. In summary, MSCs and their released components, such as exosomes, have great potential for tumor therapy, including HCC, and the transformation from preclinical research to clinical application urgently needs to be hastened.

## Conclusions and prospective

The present article discusses the recent progress in clarifying the critical roles of multipotent MSCs in HCC initiation, progression and therapy. MSCs can recruit to the liver fibrosis and HCC microenvironment, becoming the cell origin of HCC and inhibiting or promoting its progression. They and their secreted exosomes can be modified to treat HCC. We should pay additional attention to the molecular mechanisms responsible for MSC migration and attempt to enhance recruiting efficiency and improve the effect of targeted therapy. In addition, reducing the malignant transformation and growth stimulation of MSCs in HCC-targeted therapy will accelerate clinical application and a novel cell-free therapy using MSC-secreted exosomes, might constitute a compelling alternative in future research.

## Abbreviations

aHSCs: Activated HSCs
AMF: Autocrine motility factor
ANG1: Angiogenin1
bFGF: Basic fibroblast growth factor
BMDCs: Bone marrow-derived cells
C1q: Complement component 1 subcomponent q
CAFs: Cancer-associated fibroblasts
CB1: Cannabinoid receptor 1
CCM: Cell-conditioned media
CFU-F: Fibroblast colony-forming unit
cHCC-CC: combined hepatocellular-cholangiocarcinoma
CM: Conditioned media
c-MET: Mesenchymal-epithelial transition factor
CRAd: Conditionally replicative adenovirus
CSCs: Cancer stem cells
CSCs: Cancer stem-like cells
CTL: Cytotoxic lymphocyte
DCs: Dendritic cells
DR5: Death receptor 5
ECM: Extracellular matrix
ECs: Endothelial cells
EGFR: Epidermal growth factor receptor
EMT: Epithelial-mesenchymal transition
EpCAM: Epithelial cell adhesion molecule
EpICD: Intracellular domain of EpCAM
FGF: Fibroblast growth factor
hBM-MSCs: Human bone marrow-derived MSCs
HBV: Hepatitis B virus
HCC: Hepatocellular carcinoma
HGF: Hepatocyte growth factor
HIF-1: Hypoxia inducible factor1
hMSCs: Human MSCs
HNF4α: Hepatocyte nuclear factor 4α
HSCs: Hepatic stellate cells
HSV-TK: Herpes simplex virus-thymidine kinase
ICC: Intrahepatic cholangiocarcinoma
IDO: Indoleamine 2,3-dioxygenase
IFN-β: Interferon-β
IGF: Insulin growth factor
IL-1: Interleukin-1
IL-12: Interleukin-12
IL-6: Interleukin-6
IL-8: Interleukin-8
ISCT: International Society for Cellular Therapy
LCSCs: Liver cancer stem cells
MCP1: Monocyte chemoattractant protein1
MDSCs: Myeloid-derived suppressor cells
MSC-IFNβ: MSCs transfected with interferon beta
MSCs: Mesenchymal stromal cells
MV-NIS: Oncolytic measles virus encoding NIS
MVs: Microvesicles
NASH: Nonalcoholic steatohepatitis
NIS: Sodium iodide symporter
OPN: Osteopontin
PDGF: Platelet-derived growth factor
PDGFR: Platelet-derived growth factor receptor
PD-L1: Programmed death ligand-1
PEDF: Pigment epithelial-derived factor
PGE2: Phenyl glycidyl ether2
SDF1: Stromal cell-derived factor1
SHH: Sonic Hh
SIP: Sphingosine 1-phosphate
TAFs: Tumor-associated fibroblasts
TAMs: Tumor-associated macrophages
TCM: Tumor-conditioned media
TECs: Tumor endothelial cells
TGF-β: Transforming growth factor-beta
TIMP-1: Tissue inhibitor of metalloproteinase-1
TME: Tumor microenvironment
TNF-α: Tumor necrosis factor-alpha
TRAIL: Tumor necrosis factor-related apoptosis-inducing ligand
Tregs: Regulatory T cells
VEGF: Vascular endothelial growth factor
VEGFR: Vascular endothelial growth factor receptor
VM: Vasculogenic mimicry
α-SMA: Alpha- smooth muscle actin
